# Poison of Burning Charcoal

**Published:** 1855-03

**Authors:** 


Poison of Burning Charcoal.—The danger of placing ignited charcoal in a closed room was thrillingly illustrated in the family of Mr. Wm. Day, residing in Danbury, Conn., on Tuesday. Two young children were placed in bed at an early hour in the evening, and a vessel containing coal was left in the centre of the room, through a misapplied solicitude for their comfort. Before the hour for retiring of the family, they were startled by the sounds of agony proceeding from the room occupied by their children, and upon hastening to them, they were found nearly suffocated with gas. By this timely rescue, and a vigorous application of restoratives, they were both saved from a horrible death.

